# The use of tobacco in online trailers of top-grossing movies

**DOI:** 10.18332/tpc/110008

**Published:** 2019-07-01

**Authors:** Christopher M. Seitz, Matthew Craine, Josh Yates

**Affiliations:** 1 Department of Health and Exercise Science, Appalachian State University, Boone, United States

**Keywords:** tobacco, smoking, movies, policy, prevention

## Abstract

**INTRODUCTION:**

Images of tobacco use in movies have been studied extensively, showing that smoking in movies can cause the initiation of smoking among young people. Tobacco use in movie trailers, however, has not been monitored to the extent of full-length films. As such, the purpose of this study was to assess how many online trailers from top-grossing movies contain images of tobacco use, and to estimate audience exposure to those depictions.

**METHODS:**

A total of 964 trailers from the top-grossing movies of 2010–16 were located on YouTube and analyzed for the number of tobacco ‘incidents’ (tobacco went off screen and on screen, a different actor had tobacco, or a new scene contained tobacco). Audience exposure was measured through ‘impressions’, by multiplying each trailer’s number of incidents by the number of times the trailer was viewed.

**RESULTS:**

From 2010 to 2016, the trailers from top-grossing movies increased in: total per cent of tobacco incidents (16% in 2010 vs 21% in 2016), total number of incidents (69 in 2010 vs 102 in 2016), and tobacco impressions (89 million in 2010 vs 725 million in 2016).

**CONCLUSIONS:**

A considerable number of online movie trailers contain incidents of tobacco use, with a noteworthy exposure to those incidents. As such, tobacco use in movie trailers should be monitored to the same extent as full-length movies, and public health professionals should advocate for age-verification restrictions to movie trailers that contain tobacco incidents and that anti-smoking advertisements be shown before trailers containing any incidents.

## INTRODUCTION

Images of tobacco use in movies are a major public health concern. The Surgeon General reports that depictions of smoking in movies have a causational relationship with the initiation of smoking among young people^1^. Regardless, tobacco use in movies has continued. Of the 143 top-grossing movies in 2016, 59 (41%) contained images of tobacco use^2^.

Prior research suggests that movie trailers may also have an impact on viewers with regard to tobacco use. The Hanewinkel^3^ experiment with over 1000 German adolescents found that a 3- seconds depiction of smoking during a trailer impacted the audience’s views of the character’s attractiveness^3^. A different study found that from 2001 to 2002, 14% of televised movie trailers contained smoking imagery, which was watched 270 million times by 95% of all youths aged 12–17 years at that time in the United States^4^.

Given the potential impact of tobacco use in movie trailers^1-4^, there is a need to investigate tobacco use in trailers that are delivered online. The second most popular website in the world is YouTube^5^, a website that allows people to share and watch videos. YouTube has 1.9 billion users per month, and over one billion hours of video are watched per day^6^. Previous research has studied the presence of pro-tobacco videos located on YouTube^7^; however, movie trailers on the website have yet to be studied. As such, the goals of this study were to: assess the proportion of trailers from top-grossing movies located on YouTube that contain images of tobacco use, compare those findings to the trailers’ full-length films, and estimate how many people were exposed to depictions of tobacco use in online movie trailers.

## METHODS

The methods of this study were replicated from previous studies that analyzed smoking in movies^2,8^. The sample of movies were the 10 top-grossing movies of each week throughout an entire year. Other studies have used this sampling frame, as movies ranked amongst the 10 top-grossing movies, for one week or more, tend to make up 96% of ticket sales^2^. In order to compare smoking in movie trailers to the smoking found in their respective full-length films, the movies were selected from the years 2010 to 2016, which were studied previously by Tynan et al.^2^. The list of top-grossing movies was located through a searchable online database, which is maintained by ‘Smokefree Movies’ at the University of California, San Francisco (UCSF), Center for Tobacco Control^9^. Using the database, we assumed that movie trailers would not have smoking imagery if their full-length movie was categorized by Smokefree Movies as not containing smoking imagery.

Two researchers were then trained in how to determine^2,8^ a tobacco ‘incident’, which was counted each time: 1) a tobacco product went off screen and then came back on screen, 2) a different actor was shown with a tobacco product, and 3) a scene changed and the new scene contained the use or implied use of a tobacco product. Tobacco products included cigarettes, electronic cigarettes, cigars, pipes, hookah, and smokeless tobacco^2^. Afterwards, the researchers independently analyzed each trailer and recorded their findings in a document. Differences in the researchers’ analyses were resolved by a third researcher, who independently watched the trailers in question^2^.

The final number of tobacco incidents was then used to calculate online movie trailer tobacco ‘impressions’, which is ‘one person seeing one tobacco incident one time’^2^. Impressions were calculated by multiplying the number of incidents in each trailer by the number of times the trailer was watched on YouTube, known as ‘views’. The number of views for each trailer was recorded on 1 February 2019. The trailers were located by searching for the title of the movie. Only official trailers from the movie companies’ YouTube accounts were used in the study. If a trailer was not available through a movie company account, the official trailer posted through the accounts of Fandango or YouTube Movies were used.

The number of incidents and impressions were categorized by year and by the ratings provided by the Motion Picture Association of America (G, PG, PG-13, R). The results were then compared to the incidents reported by Tynan et al.^2^ regarding the trailers’ full-length films from 2010–16.

## RESULTS

The proportion of trailers from top-grossing movies that had tobacco incidents increased from 2010 to 2016. Specifically, of the 964 trailers included in the study, incidents increased from 16% in 2010 to 21% in 2016 (Table 1). The total number of incidents in movie trailers also increased from 2010 to 2016 (Table 2), which was similar to the increase in incidents in the full-length films^2^. The incidents in trailers of G and PG movies did not increase. However, the number of incidents increased in both R and PG-13 trailers, with the most dramatic increase being within PG-13 trailers. Of all R-rated movies that contained incidents, only six asked for age verification in order to view the trailer.

**Table 1:** The number of trailers, n (%), containing any incidents and the total number of impressions from 2010 to 2016

*Measurement*	*2010*	*2011*	*2012*	*2013*	*2014*	*2015*	*2016*
Trailers with incidents, n (%)	23 (16)	22 (16)	33 (23)	31 (22)	26 (19)	31 (21)	31 (21)
Impressions*	89	103	293	422	429	389	725

* Impressions are reported in millions.

**Table 2:** The number of tobacco incidents in online trailers of the top-grossing movies from 2010 to 2016

*Rating*	*2010*	*2011*	*2012*	*2013*	*2014*	*2015*	*2016*	*Total*
G/PG	0	4	0	1	2	1	0	8
PG-13	23	24	43	36	46	28	43	243
R	46	38	89	96	61	54	59*	443
**Total**	69	66	132	133	109	83	102	694

* In 2016, there was one ‘not rated’ movie trailer containing one incident, which was combined with the R-rated movie trailers.

The incidents per trailer ranged from 1 to 25 (Mean=3.5; Median=2; SD=3.6). Views per trailer ranged from 14663 to 42348150 (Mean=3926420; Median=1755537; SD=6117000). The number of impressions from trailers of top-grossing movies increased from 89 million in 2010 to 725 million in 2016 (Table 1).

## DISCUSSION

The findings of the study are noteworthy. A considerable proportion of trailers for top-grossing movies on YouTube contain incidents of tobacco use, and a large number of people are exposed to those incidents. Although movie trailers are much shorter compared to their associated full-length movies, they are still reaching millions of people online, and potentially influencing people in terms of tobacco use.

The study results have at least two important implications for the field of tobacco control. First, tobacco use in movie trailers should be monitored to the same extent as full-length movies. Currently, UCSF’s Smokefree Movies database only lists the tobacco use found in full-length movies. Given the potential impact of movie trailers, it would be worthwhile to observe trailers in addition to their full-length movies and maintain data regarding images of tobacco use.

Second, health professionals should advocate for YouTube policy changes. This would include a change in its policy to require users to verify their age to watch movie trailers that depict any use of tobacco. Given YouTube’s lack of age-verification requirements to watch R-rated trailers (only four R-rated movies required age verification for this study), it is possible for children and adolescents to watch those trailers without any obstacle. This is alarming, as trailers for R-rated movies tend to contain more tobacco incidents than those of other ratings. Regardless, trailers for movies of all ratings, especially PG-13 movies, contain tobacco incidents and should also require age verification. Moreover, based upon the recommendations of the World Health Organization, YouTube should require that strong anti-smoking advertisements be shown before all trailers that include any tobacco incidents^10^.

There are also opportunities for further research on this topic. First, there is a need for additional evidence regarding the impact of trailers on actual tobacco use behavior. Thus far, the only study on this topic was the Hanewinkel^3^ experiment that studied the impact of a brief smoking scene in a movie trailer on adolescent attitudes. Second, there is a need to investigate the age of those who are watching online movie trailers, as it is unknown which types of trailers (G/PG, PG-13, R) adolescents tend to watch online. Until then, one could safely assume that a large number of impressions come from adolescents, as YouTube is the second most popular website in the world^5^, and since 78% of YouTube users watch trailers to decide if they want to watch a full-length movie^11^.

There are limitations to this study. First, several different YouTube accounts contain the same movie trailers. Since we only analyzed trailers shown by movie companies, the total impressions would have been greater had we combined the views of all accounts. Second, as stated previously, YouTube does not have information regarding the age of those who watch videos, so it is not possible to determine how many adolescents viewed the trailers. Finally, the study findings are not generalizable to all movie trailers, since the study sample only included trailers of top-grossing movies. Moreover, the trailers in this study were of Hollywood blockbuster movies from the United States. However, it is worth noting that movies from the United States consistently account for over 60 per cent of all ticket sales across the globe^10^, meaning that the findings from this study are relevant internationally.

## CONCLUSIONS

It is well known that images of tobacco use in full-length movies have a causational relationship with the initiation of smoking among young people. Although the prevalence and exposure of tobacco use in movies is continually monitored by public health professionals, tobacco use in online movie trailers has not been extensively researched. As a result of this study, we now know that a considerable number of online movie trailers contain incidents of tobacco use, and that a large number of people are exposed to those incidents.
